# On the Suitability of Data Augmentation Techniques to Improve Parkinson’s Disease Detection with Speech Recordings

**DOI:** 10.3390/diagnostics16030498

**Published:** 2026-02-06

**Authors:** Cristian David Ríos-Urrego, Tulio Andrés Ruiz-Romero, David Puerta-Lotero, Daniel Escobar-Grisales, Juan Rafael Orozco-Arroyave

**Affiliations:** 1 GITA Lab, Faculty of Engineering, University of Antioquia, Medellín 050010, Colombia; cdavid.rios@udea.edu.co (C.D.R.-U.); tulio.ruiz@udea.edu.co (T.A.R.-R.); david.puertal@udea.edu.co (D.P.-L.); daniel.esobar@udea.edu.co (D.E.-G.); 2 LME Lab, University of Erlangen, 91054 Erlangen, Germany

**Keywords:** Parkinson’s disease, speech analysis, data-augmentation, classification, independent evaluation

## Abstract

**Background:** Parkinson’s disease (PD) is a neurodegenerative disorder that affects millions of people worldwide. Speech analysis has emerged as a non-invasive tool for automatic PD detection; however, the scarcity and homogeneity of available datasets often limit the generalization capability of machine learning models, motivating the use of data augmentation strategies to improve robustness. **Methods:** This study presents a data augmentation-based methodology for speech-based classification between PD patients and healthy control subjects. A deep learning model trained from scratch on Mel spectrograms is evaluated using augmentation techniques applied at both the waveform and time–frequency levels. Multiple training and model selection strategies are analyzed and model performance is assessed through internal validation as well as using an independent dataset **Results:** Experimental results show that carefully selected data augmentation techniques improve classification performance with respect to the non-augmented counterpart, achieving gains of up to 3% in accuracy. However, when evaluated on an independent dataset, these improvements do not consistently translate into better generalization. **Conclusions:** These findings demonstrate that, while data augmentation can effectively enhance model performance within a single dataset, this apparent robustness is not sufficient to guarantee generalization on independent speech corpora for PD detection.

## 1. Introduction

Parkinson’s disease (PD) is the second most common neurodegenerative disorder worldwide, affecting around 1% of people over 60 years [1]. Motor and non-motor alterations are commonly observed in PD patients [2]. In fact, speech disturbances have been reported as biomarkers in prodromal and early stages of PD [3,4]. Early diagnosis is crucial for implementing therapeutic strategies that mitigate the negative impacts of motor and non-motor symptoms’ progression. However, clinical assessment still relies heavily on expert observation of overt motor signs, often resulting in late diagnoses when significant neurological damage has already occurred [5]. This situation highlights the need for objective, accessible and non-invasive tools to complement traditional diagnostic procedures. In this context, voice has been suggested as a potential biomarker for early detection of PD. Between 70% and 90% of patients develop speech impairments during the course of the disease [6], including monopitch, articulatory imprecision, reduced vocal intensity and fluency disturbances [7]. These alterations are associated with hypokinetic dysarthria, which is one of the main characteristics of PD speech. It is known that they may appear even before other motor symptoms become clinically evident [8]. This makes speech a potentially valuable biomarker for early detection and unobtrusive monitoring of PD.

From a machine learning (ML) perspective, speech-based PD detection has shown promising results in controlled experimental settings. However, its real-world applicability strongly depends on the ability of models to generalize to unseen data. A major challenge in this area is the limited availability of speech datasets. Existing corpora are often small and recorded under different conditions. As a result, performance measures reported in the literature, commonly based on cross-validation, may be overly optimistic and not fully representative of real clinical scenarios. In response to these limitations, data augmentation has been increasingly used to enhance training diversity and robustness, although its current impact on cross-database generalization in speech-based PD detection remains unclear.

Within this context, PD detection from speech has been widely explored using both traditional ML and deep learning (DL) approaches. ML-based systems typically rely on handcrafted acoustic representations such as Mel Frequency Cepstral Coefficients (MFCCs), wavelet-based features, and perturbation measures, often combined with descriptors targeting specific speech dimensions including articulation, phonation, prosody, and phonemic identifiability [9,10,11,12,13]. These features are commonly paired with classical classifiers such as support vector machines, random forests, k-nearest neighbor, or neural network models, achieving competitive performance under controlled recording conditions [14,15]. More recently, DL-based approaches have gained attention due to their ability to learn discriminative representations directly from raw speech signals or time–frequency representations. Convolutional neural networks (CNNs) trained on spectrogram-based inputs have been widely adopted to capture pathological spectro-temporal patterns associated with PD [16,17,18]. In addition, hybrid architectures combining CNNs with recurrent layers have been proposed to model long-term temporal dependencies in pathological speech [19,20,21]. Beyond task-specific architectures, representation learning approaches based on auto-encoders and, more recently, self-supervised and foundation speech models such as Wav2Vec, HuBERT, and WavLM have demonstrated strong potential by providing high-level embeddings that improve robustness and generalization across speakers, languages, and recording conditions [22,23,24]. Despite these advances, speech-based PD detection systems are still largely constrained by the limited size and diversity of available datasets, which often restrict model generalization and increase the risk of over fitting.

Data augmentation has been increasingly explored in recent studies on PD speech as a practical strategy to enrich training data and improve robustness. Recent works have investigated waveform-level augmentation, including additive noise, temporal perturbations, and generative approaches based on adversarial learning [25,26]. In parallel, time–frequency transformations such as spectrogram masking and voice-specific augmentation techniques focused on pathological speech characteristics have also been proposed [27]. These approaches have been combined with deep learning architectures, including more recent transformer-based models [28]. While these methods have reported performance improvements under controlled experimental settings, most evaluations rely on internal validation protocols using a single dataset, and evidence of consistent generalization to independent speech corpora remains limited.

Driven by recent advances and the need to address the scarcity of data in pathological speech analysis, in this study, we evaluated the impact of different data augmentation strategies applied at both waveform and the time–frequency levels for speech-based PD detection. To this end, a 2D-CNN is designed and trained from scratch to discriminate between PD patients and healthy control (HC) subjects using Mel-spectrogram representations. In addition to analyzing performance gains under different training conditions, this work explicitly examines whether increasing the amount of training data through augmentation leads to more robust and better-generalizing models. To provide a realistic assessment of generalization, the proposed systems are further evaluated on an independent dataset, allowing us to analyze the extent to which data augmentation improves robustness and clinical applicability under unknown acoustic conditions.

## 2. Materials and Methods

**Figure 1:**
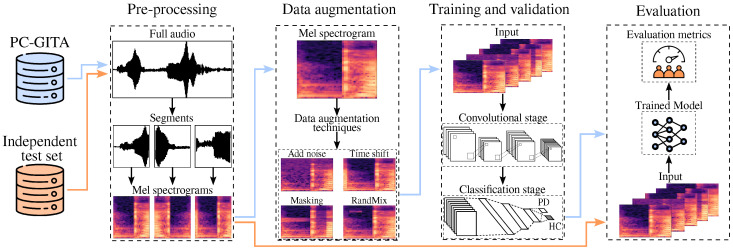
General methodology proposed in this study. Blue arrows indicate the data flow from the PC-GITA corpus, while orange arrows represent the independent test set. Elaborated by the authors.

Figure 1 presents an overview of the proposed methodology. The study is based on the analysis of speech signals from the PC-GITA corpus, used for training and validation/development, as well as from an independent test set which is employed in test exclusively. First, a pre-processing stage is applied to the full audio recordings. Each audio signal is segmented into fixed-length windows of 160 ms. These segments are subsequently transformed into Mel spectrograms that are used as the input representation. To improve the generalization capability of the model, several data augmentation techniques are applied during training, including waveform-level transformations such as noise addition and time shifting, as well as time–frequency operations such as masking and RandMix (RMi). During training and validation, the augmented Mel spectrograms from PC-GITA are used to feed a CNN responsible for automatic feature extraction followed by a fully connected layer for classification stage that discriminates between PD patients and HC subjects. Finally, the trained model is evaluated on the independent test set, and its performance is quantified using standard classification metrics like accuracy, sensitivity, specificity, and F1-score, to assess robustness and generalization under unseen conditions.

### 2.1. Data

This study is based on two speech datasets with complementary roles. The PC-GITA corpus [29] is primarily used for training and validation purposes, enabling the development and assessment of the proposed data augmentation techniques. Besides, an independent dataset, collected separately under non-controlled acoustic conditions, is employed to evaluate the generalization capability of the trained models on unseen data.

#### 2.1.1. PC-GITA Corpus

Models are trained and validated with recordings of the PC-GITA corpus [29], which consists of Colombian Spanish speech recordings from 100 participants from Medellín, Colombia. The corpus includes a total of 50 patients diagnosed with PD and 50 HC subjects matched by age, sex, and education level. All recordings were sampled at 44.1 kHz and were obtained under controlled noise conditions in a sound-proof booth and using high-fidelity audio equipment. We considered recordings of diadochokinetic (DDK) speech tasks involving the rapid repetition of syllabic sequences such as /pa/, /ta/, /ka/, /pa-ta-ka/, /pa-ka-ta/, and /pe-ta-ka/. During these tasks, participants were instructed to take a breath and continuously repeat the target syllables in a single expiration. Beginning and end parts of all recordings were manually segmented to guarantee that only relevant speech frames were included. This segmentation guarantees that long pauses are not present in the recordings. Additionally, the process allows capturing continuous articulatory and motor speech patterns that are particularly relevant to speech production and its alterations in PD.

Alongside recordings, the corpus has clinical information for all patients, including scores of the MDS-UPDRS-III (Movement Disorder Society—Unified Parkinson’s Disease Rating Scale, Part III) [30] administered by an expert Neurologist. This scale subjectively evaluates motor aspects such as rigidity, tremor, bradykinesia, posture, gait, facial expression, speech, and others. Demographic and clinical characteristics of the participants are presented in Table 1.

#### 2.1.2. Independent Test Set

The independent dataset comprises 40 subjects, including 20 individuals diagnosed with PD and 20 HC participants. The PD group consisted of 9 men and 11 women, with ages ranging from 29 to 83 years (mean = 61.3 ± 14.3). All patients were clinically assessed by the same experienced neurologist who evaluated the patients in the PC-GITA Corpus, also following the MDS-UPDRS-III scale. Resulting scores are between 9 and 106 (mean = 40.1 ± 22.7). The healthy control group included 11 males and 9 females aged between 49 and 78 years (mean = 62.6 ± 10). None of the HC participants showed evidence of neurological impairment or movement-related disorders.

All participants are native Colombian Spanish speakers. Notice that this dataset is independent from PC-GITA. Speech recordings were acquired at a sampling rate of 16 kHz. Although participants from the PC-GITA dataset performed six DDK tasks, this test set includes only recordings of the /pa-ta-ka/ DDK task.

### 2.2. Pre-Processing

A unified pre-processing pipeline was applied to both the PC-GITA corpus and the independent test set to ensure comparable input representations. First, all recordings were amplitude-normalized to reduce irrelevant loudness differences among speakers and sessions. Then, signals were converted to a standard format (single-channel/mono) and resampled to 16 kHz when needed, avoiding spectral inconsistencies due to different sampling rates. After standardization, each recording was segmented into temporal windows of 160 ms with 50% overlap. This window length was selected to capture temporal patterns associated with articulatory dynamics. In the context of diadochokinetic tasks, this temporal scale allows the representation of co-articulatory effects and syllabic transitions that are known to be affected in PD [31]. The use of 50% overlap ensures sufficient temporal coverage, guaranteeing that relevant acoustic events are fully represented in at least one segment without introducing discontinuities in the learned representations. For each segment, a Mel spectrogram was computed as the network’s input. Spectrograms were obtained using a short-time Fourier transform (STFT) with an FFT window length of 256 samples and a hop length of 64 samples. The magnitude spectra were then projected onto 80 Mel bands up to a maximum frequency of 8000 Hz, and converted to a logarithmic (dB) scale to emphasize energy differences across spectral regions.

### 2.3. Data Augmentation Techniques

**Figure 2:**
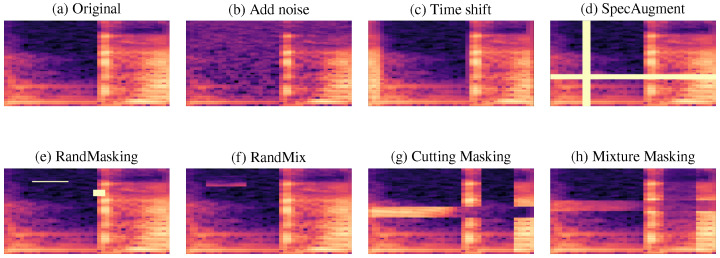
Example of Mel spectrograms obtained from the same speech sample: (**a**) original representation, (**b**) additive noise, (**c**) time shifting, (**d**) SpecAugment (time–frequency masking), (**e**) RandMasking (multiple random masks), (**f**) RandMix (block replacement from another sample), (**g**) Cutting Masking (block-wise cut-and-paste), and (**h**) Mixture Masking (block-level mixing). Elaborated by the authors.

To address data scarcity and improve model robustness, we considered seven data augmentation techniques applied at two complementary levels: waveform (operating on the audio signal directly) and time–frequency transformations (applied to the resulting Mel spectrograms). These techniques aim at incorporating realistic variability to the audio samples while preserving the underlying clinical characteristics of the original sample. Figure 2 illustrates the effect of each augmentation strategy on the same input. Details are presented in the following subsections.

#### 2.3.1. Waveform-Level Transformations

Waveform-level augmentation techniques operate directly on the raw audio signal prior to the spectrogram computation. Additive noise simulates mild acquisition perturbations by injecting a low-amplitude noise sequence into the signal [32],

(1) $$x^{\prime }\left(t\right)=x\left(t\right)+\alpha \;n\left(t\right),$$

where n(t) denotes a noise signal and α controls its intensity. This transformation encourages the model to learn discriminative speech patterns that are robust to background interference. The corresponding effect on the Mel spectrogram is illustrated in Figure 2b, where relevant spectral structures are preserved despite increased local variability.

Time shifting is another method typically used to displace the waveform by a small fraction of its duration [33],

(2) $$x^{\prime }\left(t\right)=x\left(t-\tau \right),$$

where τ denotes the applied temporal shift. The effect of this method is observed in the phase; however, although this operation does not modify the spectral content of the signal, it introduces temporal variability and therefore reduces the model’s sensitivity to absolute time alignment. The impact of this transformation in the time–frequency domain is shown in Figure 2c.

#### 2.3.2. Time–Frequency Transformations

After computing the Mel spectrograms, we applied a set of masking and block-based transformations that modify localized time–frequency regions while preserving the global structure of the representation. Initially, SA introduces rectangular masks along time and/or frequency axes, forcing the network to rely on complementary acoustic cues rather than specific localized regions. As shown in Figure 2d, this strategy effectively removes contiguous temporal and spectral regions, improving invariance to missing or corrupted information [34].

RM extends SA by applying multiple randomly positioned ‘masks of varying sizes within the same spectrogram. This increases the diversity of occluded regions and promotes learning more robust representations [35]’. An example of RM is shown in Figure 2e. Beyond masking, we evaluated block replacement and mixing strategies that explicitly combine information from different samples. RMi replaces randomly selected blocks of a spectrogram with blocks taken from another sample, introducing structured local variability while preserving global context (Figure 2f) [36].

Cutting Masking (CM) performs a cut-and-paste operation in which specific time–frequency regions of a target spectrogram are replaced by corresponding regions from another sample in the same mini-batch [37]. This operation introduces abrupt local changes while maintaining realistic spectral patterns, as illustrated in Figure 2g.

Finally, Mixture Masking (MM) combines selected regions from two spectrograms through weighted averaging, generating smoother interpolated representations that blend acoustic characteristics from both samples [38]. Figure 2h shows an example of this operation.

### 2.4. Training and Validation

During the training and validation stages, a two-dimensional 2D-CNN was employed to model the Mel spectrogram representations. The convolutional module acts as a feature extractor, while a subsequent fully connected stage performs the classification between PD patients and HC subjects.

#### 2.4.1. Convolutional Stage

The convolutional stage is designed to automatically learn discriminative time–frequency patterns from Mel spectrograms. In this framework, the input spectrogram is treated as a two-dimensional representation with one or more channels over which a set of learnable filters is applied. Each convolutional layer performs local operations that capture short-term spectral and temporal dependencies by sliding kernels across the input and generating feature maps that emphasize relevant acoustic structures.

By stacking multiple convolutional layers, the network progressively builds hierarchical representations. Early layers tend to capture low-level patterns such as spectral edges or local energy variations, whereas deeper layers integrate these cues into more abstract and task-relevant features. This hierarchical modeling capability allows the CNN to effectively represent complex speech characteristics.

To improve robustness and computational efficiency, convolutional layers are interleaved with pooling operations, therefore reducing the spatial resolution of the feature maps by aggregating local responses, which helps in controlling model complexity, provides a degree of invariance to small spectral or temporal shifts, and mitigates over-fitting.

#### 2.4.2. Classification Stage

After the convolutional and pooling operations, the resulting feature maps are flattened and passed through fully connected layers. The final fully connected layer acts as a classifier, with the number of output neurons corresponding to the number of target classes. This classification stage transforms the compact feature representation into class probabilities, enabling the network to discriminate between PD and HC subjects. By separating feature extraction and classification into distinct stages, the model benefits from both localized pattern learning and global decision-making.

### 2.5. Model Evaluation

The evaluation of deep learning models allows estimating their generalization capability when exposed to unseen data. While cross-validation is widely adopted to obtain statistically robust performance estimates during training and validation, it does not define a unique final model to be evaluated. Different reasonable model selection criteria can lead to different generalization behaviors. For this reason, in this work we explicitly analyze multiple model selection and training strategies to provide a comprehensive and unbiased assessment of model performance.

First, a train 100% strategy was employed, where the model was trained using the entire PC-GITA dataset and subsequently evaluated on the independent test set. This configuration represents an upper-bound scenario, in which the model benefits from the maximum amount of training data, providing insight into its best achievable performance. Second, a holdout 70/30 strategy was applied. In this setting, 70% of the available data was used for training and the remaining 30% was reserved for internal validation. After training, the resulting model was evaluated on the independent test set. This strategy reflects a more realistic scenario, where the model must generalize effectively with limited training data.

Third, different cross-validation-based approaches were explored to analyze model stability and variability across different data partitions. The PC-GITA dataset was divided into multiple folds, and the model was trained and validated iteratively across these folds. Within this framework, two complementary model selection criteria were considered. The best model cross-validation strategy selects the model instance that achieved the highest validation performance among all folds. This approach represents a best-case scenario and provides insight into the maximum performance attainable under favorable training conditions. Conversely, the mean model cross-validation strategy selects the model whose validation performance is closest to the average performance across all folds. This criterion yields a less biased and more representative model, as it better reflects the expected behavior of the system and accounts for performance variability induced by different training partitions.

By combining these different training and model selection strategies, this evaluation framework provides a comprehensive analysis of model performance across optimistic, realistic, and statistically stable scenarios. This evaluation is essential to better understand the generalization properties and robustness of the proposed approach when applied to independent and unseen data.

## 3. Experiments and Results

This section presents the experimental setup and results obtained with the proposed CNN-based approach. The experiments are organized into three main stages. In the first one, the design and selection of the CNN architecture are conducted using the PC-GITA corpus only, without applying any data augmentation techniques. This analysis aims at identifying a robust baseline. In the second stage, the selected CNN architecture is evaluated under different data augmentation strategies to analyze their impact on classification performance and model robustness. Finally, in the third stage, the best-performing configurations obtained from the baseline and data augmentation experiments are evaluated on an independent dataset. This stage focuses on assessing the generalization capability of the proposed approach under unseen conditions, using the different validation strategies described in Section 2.4. All computational experiments were carried out on a workstation equipped with an NVIDIA Quadro RTX 5000 GPU. The model development was implemented using the PyTorch framework (version 2.6.0, CUDA 12.4).

For all experiments reported in Section 3.1 and Section 3.2, which are based on the PC-GITA corpus, a subject-independent stratified five-fold cross-validation strategy was employed. In contrast, the experiments in Section 3.3 use the independent dataset to assess the generalization performance of the trained models. For each experimental configuration, performance was quantified using four evaluation metrics: accuracy, sensitivity, specificity, and F1-score. Although both datasets used in this paper are balanced in age and gender, these metrics provide complementary evaluation on overall performance and class-dependent behavior which is relevant in clinical classification tasks.

### 3.1. CNN Architecture Design

The performance of CNN architectures with different depths was evaluated upon recordings of the PC-GITA corpus without applying any data augmentation techniques. CNN architectures with 2, 3, 4, and 5 convolutional layers were considered. Kernel size and pooling configuration were kept fixed across all experiments to ensure a controlled evaluation of the impact of data augmentation and validation strategies. The main objective of these analyses was to identify a baseline architecture with balanced performance. The resulting model was later used to assess the impact of data augmentation strategies. Table 2 summarizes the performance obtained for each architecture. Mean and standard deviation are reported across folds.

As shown in Table 2, the CNN with four convolutional layers achieves the most balanced performance across metrics. Although the five-layer CNN exhibits the highest sensitivity, its low specificity indicates a high false positive rate. In contrast, the CNN-4 architecture presents a better trade-off between sensitivity, specificity, and F1-score, making it the most suitable candidate as the baseline model for subsequent data augmentation experiments.

The selected baseline architecture consists of a CNN implemented in PyTorch, designed to process single-channel time–frequency representations of 80×41 and perform binary classification between PD and HC subjects. The network is composed of four convolutional blocks followed by three fully connected layers. Each convolution uses a 3×3 kernel with padding 1, followed by ReLU activation and 2×2 max pooling. The input dimensionality of the first fully connected layer (320) corresponds to the flattened output of the last convolutional block after pooling. A summary of the architecture is reported in Table 3.

### 3.2. CNN with Data Augmentation

This section presents results obtained when applying different data augmentation techniques to the CNN-based model for PD detection. The main objective is to evaluate the effect of individual and combined augmentation strategies. All augmentation techniques considered in these analyses were applied symmetrically to both PD patients and HC subjects. As a result, for each augmentation scenario, the effective size of the training dataset was doubled, ensuring a fair comparison across configurations.

Table 4 summarizes the results obtained when individual data augmentation techniques were applied. The baseline model trained without data augmentation achieved an acceptable performance (accuracy of 0.81), serving as a reference point to quantify the effect of each strategy. Among the evaluated techniques, CM yielded the best overall performance, improving the baseline accuracy by approx. 2%. Moreover, this configuration exhibited a balanced trade-off between sensitivity and specificity while reducing performance variability across folds, indicating improved model stability. Similarly, RM produced consistent and uniform results across all metrics, suggesting reliable behavior and robustness to different data partitions. On other hand, SA achieved the highest sensitivity, indicating a strong ability to correctly identify PD patients. However, this approach also exhibits an increased number of false positives. In contrast, MM and RM did not consistently outperform the baseline model, suggesting that not all augmentation strategies are equally beneficial in this task.

Overall, these results illustrate that individual augmentation techniques influence model performance in different ways, affecting not only accuracy but also variability and sensitivity–specificity balance. This analysis provides important criteria for selecting augmentation strategies according to the clinical objectives defined for the system.

In addition to individual techniques, Table 5 indicates the results obtained with several combinations of data augmentation methods. Notice that while some combinations improved specific metrics, not all configurations led to superior overall performance. In particular, certain combinations negatively affected sensitivity and F1-score, suggesting that the simultaneous application of multiple masking strategies may introduce excessive distortion.

Among all evaluated combinations, the SA + CM configuration achieved the best overall performance, reaching an accuracy of 0.84±0.04 and the same values for the F1-score. This configuration maintained a strong balance between sensitivity and specificity, outperforming both the baseline model and all individual augmentation strategies. In addition, it consistently reduced performance variability across metrics, resulting in the most robust model trained on the PC-GITA dataset. Other combinations exhibited unbalanced values. For instance, the CM + MM combination favored specificity at the expense of sensitivity, while combinations involving RM failed to surpass the baseline performance. These findings indicate that combining augmentation techniques does not necessarily yield positive effects; additionally, a careful selection of complementary strategies is necessary.

Overall, the results above demonstrate that data augmentation enhances both the effectiveness and robustness of CNN-based models for speech-based PD detection. However, they also highlight that performance gains are highly dependent on which augmentation strategy or combination is used. A careful selection of techniques results in more reliable improvements, therefore constituting a strong candidate for deploying PD detection systems.

### 3.3. Evaluation in an Independent Test Set

This section evaluates the generalization capability of the proposed model using an independent dataset, representing a realistic validation scenario. The analysis focuses on the comparison of three configurations: (i) the baseline model without data augmentation; (ii) the model trained with CM; and (iii) the model trained with the best augmentation combination (CM + SA). The results under different validation strategies are summarized in Table 6.

Overall, the results reveal a consistent degradation in performance across configurations, highlighting the strong sensitivity of the models to dataset mismatch and evidencing a possible over-fitting effect. Across all validation strategies, both the baseline and data-augmented models exhibit extreme behaviors characterized by either very high sensitivity coupled with poor specificity or vice versa. This instability suggests that, despite the apparent performance gains observed on the PC-GITA corpus, the learned representations do not generalize robustly to unseen data recorded under unknown acoustic conditions.

In particular, models trained with data augmentation strategies including CM and its combination with SA do not consistently outperform the baseline model when evaluated on the independent dataset. While these techniques improve robustness within the training corpus, their benefits do not translate into improved generalization. Conversely, in several cases they introduce additional variability or bias toward the positive class. An exception is observed under the best model cross-validation strategy, where the baseline configuration achieves the highest accuracy with a relatively balanced trade-off between sensitivity and specificity. This result suggests that, under favorable training conditions, the baseline model is capable of learning discriminative patterns that partially generalize to unseen data. However, this behavior is not consistently replicated across other validation strategies, underscoring the difficulty of selecting a reliable evaluation protocol.

These findings indicate that increasing data diversity through the explored augmentation techniques is not sufficient to ensure generalization across datasets. While data augmentation can effectively regularize models within a single corpus, it may also introduce distortions that limit transferability to independent data. This highlights the intrinsic challenge of validating speech-based PD detection systems and reinforces the need for independent datasets when assessing real-world clinical applicability. Previous studies have only reported results of data augmentation methods within the same corpus, therefore limiting their generalizability to real clinical settings [25,39,40].

## 4. Discussion

This study analyzed the suitability of different data augmentation strategies for improving speech-based PD detection using Mel spectrogram representations and a CNN-based architecture. Augmentation techniques were applied at both the waveform and time–frequency levels and evaluated under multiple training and validation strategies. Results obtained using cross-validation on the PC-GITA corpus show that time–frequency augmentation methods, particularly SpecAugment (SA) and Cutting Masking (CM), can improve classification performance within a single dataset, increasing accuracy from 81% to 84% while reducing performance variability. These findings are consistent with previous studies reporting benefits from spectrogram-based augmentation when training and testing are performed on data drawn from the same corpus.

However, such results should be interpreted with caution. Most speech-based PD detection studies rely on recordings from a single corpus for both training and evaluation, largely due to the difficulty of collecting pathological speech data. In this work, we included an additional dataset composed of 40 previously unseen speakers, which was used exclusively for testing without further hyperparameter optimization. Under this realistic evaluation scenario, none of the evaluated augmentation strategies improved the performance of the baseline system, which achieved an accuracy of up to 80%. The comparison of multiple training and validation strategies further demonstrates that cross-validation alone is insufficient to guarantee real-world generalization. Although data augmentation can act as an effective regularizer within a single dataset, its benefits do not necessarily transfer to unseen data and may even induce overfitting, highlighting the importance of evaluation on independent datasets.

From a clinical perspective, the results of this study highlight several aspects that should be taken into account when interpreting speech-based PD detection studies. Although increasing data through augmentation may improve performance under internal validation, our findings show that these improvements do not generalize to independent datasets. This suggests that such systems may exhibit biased behavior and should be considered as supportive tools rather than standalone diagnostic solutions. In practice, relying on models that have not been properly validated using independent data can lead to overly optimistic results and high false positive rates. This raises important ethical concerns, as false alarms could negatively impact patients and clinical decision-making. Therefore, the use of independently validated models is essential to reduce the risk of misdiagnosis.

To further strengthen these findings, recent advances in speech processing have shown that transfer learning and large-scale pre-trained models can work well in clinical tasks with limited labeled data. Under certain conditions, due to the large amount of trained data, they could help with model generalization. However, reported gains from such approaches are commonly obtained using internal validation protocols, which may still overestimate real-world performance. In this study, we deliberately prioritized evaluation on an independent dataset to emphasize that generalization depends not only on model architecture or pre-training, but also on how performance is assessed. From this perspective, this work highlights the need for external validation regardless of the learning paradigm employed.

Several limitations of this study should be acknowledged. The experiments are constrained by the size and homogeneity of the available speech datasets, a common limitation in pathological speech research. Although data augmentation increased the effective size of the training data, it could not compensate for the limited demographic and pathological variability of the original corpus. Moreover, the lack of consistent improvements on an independent dataset indicates that performance gains observed under cross-validation may overestimate real-world generalization.

Future work will explore data augmentation in combination with more advanced learning paradigms, such as transfer learning and large-scale pre-trained speech models, as well as their behavior across PD subtypes and other diseases closely related to PD. Importantly, these approaches should be systematically evaluated using independent datasets to ensure reliable generalization.

## 5. Conclusions

This study investigated the use of data augmentation techniques to improve speech-based classification between PD patients and HC subjects. A CNN-based model trained on Mel spectrogram representations was evaluated using several augmentation strategies applied at both the waveform and time–frequency levels. Experimental results showed that some augmentation methods, particularly SpecAugment and Cutting Masking, can improve classification performance when evaluated using cross-validation on a single dataset, achieving accuracy gains of up to approximately 3% compared to a non-augmented baseline. However, when the same models were evaluated on an independent dataset, these improvements did not consistently translate into better generalization, and the baseline model achieved comparable or better performance. These findings lead to a key conclusion of this study: results obtained using internal validation alone can be overly optimistic, and independent test datasets are necessary to properly evaluate the real-world generalization of speech-based PD detection systems.

## Figures and Tables

**Table 1:** Demographic and clinical statistics of the PC-GITA corpus. Values are expressed as mean ± standard deviation. M: Male, F: Female. Elaborated by the authors.

Variable	PD (M)	HC (M)	PD (F)	HC (F)
Number of subjects	25	25	25	25
Age (years) *	61.3 ± 11	60.5 ± 12	60.7 ± 7	61.4 ± 7
Age range (years)	33–81	31–86	49–75	49–76
Years since diagnosis	8.7 ± 6		12.6 ± 12	
MDS-UPDRS-III	37.8 ± 22		37.6 ± 14	
Speech item (MDS-UPDRS-III)	1.4 ± 0.9		1.3 ± 0.8	

* *p*-value = 0.77 calculated through a *t* test.

**Table 2:** Performance of CNN architectures with different numbers of convolutional layers. Results reported as mean ± standard deviation. Elaborated by the authors.

Model	Accuracy	Sensitivity	Specificity	F1-Score
CNN 2 Layers	0.70 ± 0.04	0.64 ± 0.16	0.76 ± 0.10	0.67 ± 0.09
CNN 3 Layers	0.72 ± 0.02	0.78 ± 0.07	0.66 ± 0.05	0.73 ± 0.03
CNN 4 Layers	0.73 ± 0.04	0.74 ± 0.12	0.72 ± 0.07	0.73 ± 0.06
CNN 5 Layers	0.68 ± 0.12	0.82 ± 0.12	0.54 ± 0.19	0.72 ± 0.10

**Table 3:** CNN 4 layer architecture. Elaborated by the authors.

Layer	Input Channels	Output Channels	Kernel Size
Conv1 + ReLU + MaxPool	1	4	3×3
Conv2 + ReLU + MaxPool	4	8	3×3
Conv3 + ReLU + MaxPool	8	16	3×3
Conv4 + ReLU + MaxPool	16	32	3×3
Fully connected 1	320	128	–
Fully connected 2	128	64	–
Fully connected 3	64	2	–

**Table 4:** Performance results using individual data augmentation techniques. Baseline: no data augmentation. CM: Cutting Masking. MM: Mixture Masking. RM: Rand Masking. RMi: Rand Mix. SA: SpecAugment. Elaborated by the authors.

Method	Accuracy	Sensitivity	Specificity	F1-Score
Baseline	0.81±0.07	0.80±0.18	0.82±0.18	0.80±0.09
Waveform-level	0.80±0.06	0.86±0.08	0.74±0.12	0.81±0.06
CM	0.83±0.08	0.82±0.10	0.84±0.14	0.83±0.08
MM	0.79±0.05	0.76±0.10	0.82±0.12	0.78±0.05
RM	0.82±0.07	0.82±0.07	0.82±0.12	0.82±0.07
RMi	0.80±0.05	0.76±0.10	0.84±0.14	0.79±0.06
SA	0.82±0.08	0.88±0.10	0.76±0.21	0.83±0.06

**Table 5:** Performance results using combinations of data augmentation techniques. Baseline: no data augmentation. CM: Cutting Masking. MM: Mixture Masking. RM: Rand Masking. SA: SpecAugment. Elaborated by the authors.

Method	Accuracy	Sensitivity	Specificity	F1-Score
Baseline	0.81±0.07	0.80±0.18	0.82±0.18	0.80±0.09
CM + MM	0.83±0.07	0.76±0.14	0.90±0.00	0.81±0.10
RM + CM	0.78±0.07	0.80±0.13	0.76±0.19	0.78±0.06
RM + MM	0.77±0.05	0.72±0.15	0.82±0.10	0.75±0.08
RM + CM + SA	0.80±0.05	0.70±0.06	0.90±0.06	0.78±0.06
CM + SA	0.84±0.04	0.84±0.10	0.84±0.08	0.84±0.04

**Table 6:** Results of the CNN model evaluated using an independent dataset under different validation strategies. Baseline: no data augmentation. CM: Cutting Masking, SA: SpecAugment. Elaborated by the authors.

Validation Strategy	Method	Accuracy	Sensitivity	Specificity	F1-Score
Train 100%	Baseline	0.58	1.00	0.15	0.70
	CM	0.58	1.00	0.15	0.70
	CM + SA	0.68	1.00	0.35	0.75
Holdout 70/30	Baseline	0.68	0.40	0.95	0.55
	CM	0.58	1.00	0.15	0.70
	CM + SA	0.60	0.95	0.25	0.70
Best model cross-validation	Baseline	0.80	0.85	0.75	0.81
	CM	0.50	0.95	0.05	0.66
	CM + SA	0.60	1.00	0.20	0.71
Mean model cross-validation	Baseline	0.73	1.00	0.45	0.78
	CM	0.60	0.90	0.30	0.69
	CM + SA	0.70	0.85	0.55	0.74

## Data Availability

The original contributions presented in this study are included in the article. Further inquiries can be directed to the corresponding author.
